# Memory Modulation Factors in Hippocampus Exposed to Radiation

**DOI:** 10.17691/stm2021.13.4.01

**Published:** 2021-08-28

**Authors:** O.A. Krotkova, A.Y. Kuleva, M.V. Galkin, M.Y. Kaverina, Y.V. Strunina, G.V. Danilov

**Affiliations:** Senior Researcher, Rehabilitation Unit; N.N. Burdenko National Medical Research Center for Neurosurgery, Ministry of Health of the Russian Federation, 16, 4^th^ Tverskaya-Yamskaya St., Moscow, 125047, Russia; PhD Student; Institute of Higher Nervous Activity and Neurophysiology, Russian Academy of Sciences, 5A Butlerova St., Moscow, 117485, Russia; Researcher, Radiotherapy Department; N.N. Burdenko National Medical Research Center for Neurosurgery, Ministry of Health of the Russian Federation, 16, 4^th^ Tverskaya-Yamskaya St., Moscow, 125047, Russia; Junior Researcher, Rehabilitation Unit; N.N. Burdenko National Medical Research Center for Neurosurgery, Ministry of Health of the Russian Federation, 16, 4^th^ Tverskaya-Yamskaya St., Moscow, 125047, Russia; Managing Engineer; N.N. Burdenko National Medical Research Center for Neurosurgery, Ministry of Health of the Russian Federation, 16, 4^th^ Tverskaya-Yamskaya St., Moscow, 125047, Russia; Scientific Secretary; N.N. Burdenko National Medical Research Center for Neurosurgery, Ministry of Health of the Russian Federation, 16, 4^th^ Tverskaya-Yamskaya St., Moscow, 125047, Russia; Associate Professor, Neurosurgery Department; N.N. Burdenko National Medical Research Center for Neurosurgery, Ministry of Health of the Russian Federation, 16, 4^th^ Tverskaya-Yamskaya St., Moscow, 125047, Russia

**Keywords:** hippocampus, neurogenesis, memory impairment, attention, radiotherapy of parasellar meningiomas

## Abstract

Although the key scene of the hippocampus in memory processes is obvious, the specificity of its participation in information processing is far from being established. Current advanced neuroimaging enables to operate with precise morphometric parameters.

**The aim of the study** was to reveal fine memory rearrangements under mechanical impact on the hippocampus by a neoplasm and radiation exposure in the course of therapy.

**Materials and Methods:**

We used a homogeneous sample of 28 patients with parasellar meningiomas adjacent to hippocampus. In 10 patients (5 with left-sided and 5 with right-sided meningiomas), the tumor was located near the hippocampus but exhibited no mechanical effect on it. In 18 patients (10 with left-sided and 8 with right-sided tumors), the neoplasm compressed the adjacent hippocampus. The control group consisted of 39 healthy subjects. All three groups were comparable in age, education, and gender characteristics. In order to control tumor growth, the patients underwent radiotherapy when the hippocampus involuntary was exposed to a dose comparable to that in the tumor (30 sessions with a single focal dose of 1.8 Gy, total dose — 54.0 Gy).

Based on the literature data on hippocampus involved in mnestic processes, a special methodology to investigate memory was developed. Incorrect responses the subjects made when identifying previously memorized images were classified as neutralizing the novelty factor of an identified stimulus or as wrongly emphasizing its novelty.

**Results:**

At the first observation point (before radiation therapy) all groups underwent a complete standardized neuropsychological examination and performed a battery of cognitive tests. The overall results of the tests assessing attention, memory, thinking processes, and neurodynamic indicators corresponded to standard values. A mild brain compression by the tumor without brain tissue destruction was not accompanied by focal neuropsychological symptoms and deficit manifestations in the cognitive sphere. However, as early as in the first observation point, the number of “pattern separation” errors in the clinical group was significantly higher than that in healthy subjects.

The second observation point (immediately after radiotherapy) and the third observation point — 6 months after the treatment — showed that, in general, the patients’ cognitive sphere condition was not deteriorating, and in a number of parameters was characterized by positive dynamics, apparently associated with some tumor reduction due to the therapy provided. However, the distribution of errors in the original method significantly changed. When previously memorized stimuli were recognized, the errors neutralizing the novelty factor of the evaluated stimulus increased, while the number of errors with overestimating the stimuli novelty decreased.

All tendencies hypothetically (according to the published data) associated with the changes in functional activity of the hippocampus were more pronounced in the subgroup of patients with mechanical impact of the tumor on hippocampus.

**Conclusion:**

The continuous flow of impressions any person has at any moment of his activity is most likely marked by the hippocampus in a continuum “old–similar–new”. The present study has shown that mechanical impact on the hippocampus combined with radiation exposure changes the range of assessments towards the prevailing labeling “old, previously seen, already known”.

## Introduction

Current knowledge on brain arrangement of mnestic processes are related to the data on memory as the function provided by all parts of a distributed brain neural network assuming that different divisions contribute to information imprinting and preservation, their contribution being specific. Research in the sphere provides basic knowledge for further solution of memory modulation problems including those using new neurocognitive technologies. Mnestic processes are not unitary by their nature; however, they include many parameters and elements, which are differently responsive to the changes of the functional brain condition. One of the key roles is played by the hippocampus. Close connection of the hippocampus with mnestic processes was established long ago and has never been disputed. The presentation of the patient [1], who underwent hippocampus bisection in order to treat epilepsy, showed almost complete failure to memorize current impressions. Further clinical observations more than once have confirmed the data, and unilateral damage of this brain structure was reported not to result in global memory impairments, as a rule (e.g. [2]). Hippocampus impairment in animal models demonstrated behavioral alterations indicating the hippocampus functions like “comparator” filtrating fresh information by comparing it to the previous information, the storage of which is provided by other parts of the brain [3].

The capabilities of high-resolution multivoxel magnetic resonance spectroscopy enabled to take steps in understanding hippocampus functional involvement in mnestic processes. The terms — pattern separation and patterns completion — appeared to be the most popular [4]. Any impressions of current experience are compared to those of the previous experience. They can be assessed as crucially new, can be evaluated as similar, but being different in key features, or being those completely repeating the previous experience (being different in insignificant characteristics). Such assessment of impressions is likely to happen at hippocampus functional activity level.

Hypothetically, neurogenesis processes appear to participate in solving such complex task. Hippocampus is neurogenic structure — the area, where new neurons are produced from progenitor cells. The formed neurons migrating from the hippocampus are embedded in brain network parts forming new functional systems of the brain [5]. The process of differentiating patterns of information — pattern separation and pattern completion of impressions from current experience — considerably changes with age. It was proved experimentally that at elderly age compared to younger age to assess two stimuli as similar rather than identical, a significantly larger degree of difference between these stimuli is required at both: at the level of behavioral responses, and by multivoxel pattern in the hippocampus [6]. In the context of neurogenesis decreasing with age, these data also indirectly confirm the importance of the hippocampus in the analyzed phenomenology.

In the direction of these studies, a fundamentally new step could be the observation of a homogeneous sample of patients under the influence of different factors on the hippocampus.

The implementation of the conditions has become possible when following up the patients with parasellar meningiomas adjacent to the hippocampus. These extracerebral benign tumors locate at basal brain surface in close proximity to mediobasal parts of the left and the right temporal lobe. A tumor compresses the structures of “its” hemisphere without infiltrating the brain matter (without destroying it). Slow growth of such neoplasms accompanied by compensatory reconstructions is one more explanation of no marked clinical presentation in the described sampling [7, 8]. To stop tumor growth, patients undergo radiation therapy, in which the hippocampus is forced to receive a dose comparable to the dose in the tumor [9].

**The study aimed** at establishing the relation between the factors of mechanic impact on the hippocampus by a lesion, the radiotherapy effect on the hippocampus, and the changes in patients’ memory corresponding to these two factors, at different points of the longitudinal study.

## Materials and Methods

The investigation involved 39 healthy subjects (mean age — 51±21 years), among them there were 29 women (74.4%) and 10 men (25.6%), as well as 28 patients with parasellar meningiomas (mean age — 51±10 years), among them: 24 women (85.7%), 4 men (14.3%). Later, the patients with meningiomas were divided into two subgroups: those with tumors compressing the temporal lobe without the hippocampus displacement; and the patients with tumors compressing the hippocampus. All the groups were comparable in age, education, and gender characteristics. The present study was carried out in accordance with the Declaration of Helsinki (2013) and was approved by the Ethics Committee of N.N. Burdenko National Medical Research Center for Neurosurgery (Moscow, Russia).

**Figure 1:**
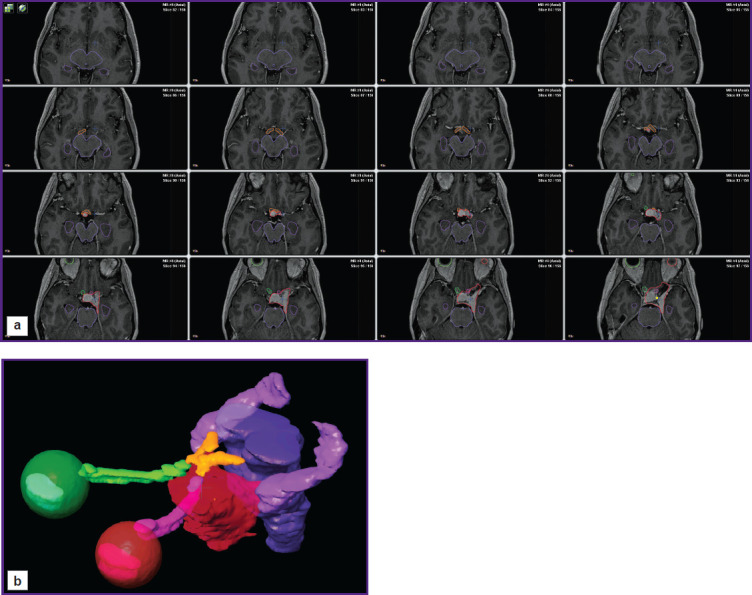
Tomogram of patient Z. from the first subgroup: (a) layer-by-layer contouring of hippocampuses (*violet contours*) and the tumor (*red contours*) on axial T1 images with contrast enhancement; (b) 3D reconstruction of the tumor and main critical structures

None of the patients with meningiomas had radiotherapy and neurosurgeries in past medical history. All cases of benign meningiomas were diagnosed based on typical clinical presentation and neuroimaging data. All patients underwent head MRI in axial view, in 3D SPGR mode (before and after contrast administration, section thickness — 1.0 mm), and in Т2 mode (before contrast administration, section thickness — 2.0 mm). Tumors and hippocampuses were contoured using a radiation planning system iPlan (Brainlab, Germany) for precise assessment of a tumor volume, its special location, and the hippocampus compression degree. Hippocampuses were contoured according to the radiotherapy protocol RTOG 0933 and the study by Chera et al. [10] on axial images, successively, manually, on each section using all available modalities. Figure 1 (a) shows the examples of the hippocampus and tumor layer-by-layer contouring.

All patients underwent stereotactic conformal radiation therapy using a photon beam according to a standard method on the linear electron accelerator (6 MeV) Novalis (Brainlab) equipped with a micro-multi-leaf collimator. The treatment was provided in the Radiotherapy Department in N.N. Burdenko National Medical Research Center for Neurosurgery (Moscow, Russia).

At the first stage, the head was immobilized using an individual thermoplastic mask. Then, under the conditions of mask fixation, topographometric helical computed tomography was performed. The data of the previous MRI and topographometric helical computed tomography were transmitted on a planning system iPlan, where they were fused and localized in coordinates of a medical device. After that, the contours of the target and critical structures were determined and dosimetric planning was carried out. An individual radiotherapy program suggested detecting an optimum relationship of a medical dose to a tumor and the radiation exposure to adjacent critical structures. After approval, the program was sent to a medical device.

The course of treatment consisted of 30 daily (except for day-offs and public holidays) radiotherapy sessions in the mode of standard fractionation, with a single basic dose of 1.8 Gy, total dose: 54.0 Gy. The radiation dose on healthy tissues (15 cm^3^) averaged 47.0±4.0 Gy. Radiation doses on 10, 30, and 50% of ipsilateral hippocampus volume, respectively, were 40.0±8.0, 29.0±8.0, and 21.0±8.0 Gy.

A day for memory testing was marked as some time point. The first point corresponded to the examination carried out before the radiation therapy. The second point corresponded to the examination performed immediately after the treatment (generally, 45 days after the first point). The third point corresponded to 6 months after radiation therapy completed.

Based on literature data on the hippocampus involvement in mnestic processes, we developed an original EAM method (eye tracking–attention–memory). A subject under investigation was successively shown on the monitor 5 stimuli and instructed “to look at them carefully and remember”. Each stimulus included three color pictures in a row (a triplet). Exposure time for a triplet was 10 s. Before the presentation and during the pauses between the triplets a research subject was shown a grey display (it was reported beforehand that “when a grey display is demonstrated one should rest doing nothing”). The total duration of the presentation was 110 s, being accompanied by recording the subject’s eye movement. Subjects received no instructions on the screen place where their gaze should settle before the stimulus material being displayed and during the pauses. Strategies of visual attention distribution were regulated only by the spontaneous activity of the participants themselves.

Ten minutes after the presentation, the procedure of free reproduction of the stimuli keep in mind was carried out. A research subject was to recall and name in any order the pictures having been demonstrated on display. The answer records were taken. In another 15 min, a subject underwent a recognition of stimulus material procedure. On a computer monitor, single pictures appeared in a pseudorandom order. Among them, there were those completely identical to the original sample, as well as those somewhat different from it in small details, color, spatial arrangement. In addition, completely new pictures appeared in a pseudorandom order. They had no relation to the original image. When a subject was shown a picture, he was to say if he had seen it before, or if he had seen a similar one, or if there had been no such a picture at all. Moreover, before the experiment, the differences in answers: “the same” and “the similar” were demonstrated using examples. The study involved the subjects who understood the meaning of these words.

Stimulus material at a recognition stage consisted of 30 pictures: 15 pictures identical to the sample; 10 pictures similar to the lateral stimuli in triplets; 5 new distracters. Three sets of stimulus material in the EAM technique in the course of a preliminary approval on healthy subjects were set equal in perception complexity and difficulty in retention. Each time, a new set of stimulus material was demonstrated to a subject under study during the experiment at three time points.

At each point, in addition to EAM testing, we performed a complete standard neuropsychological examination according to the Luriya method [11] and a battery of cognitive tests (tests from Wechsler Memory Scale, dichotic listening, a tapping test, etc.).

### Statistical analysis

The data were processed using the statistical programming language R v. 3.6.1 in IDE RStudio v. 1.2.1335. The distribution of continuous and discrete qualitative variables in the sampling was represented as the arithmetic mean and standard deviation (M±σ) for normally distributed random variables; median and quartiles (Me [Q1; Q3]) for variables with the distribution different from normal. Categorical variables are represented as an absolute number and percentage ratio (n (%)). Shapiro–Wilk test was used to test distribution normality. Mann–Whitney U test was applied to test statistical hypotheses on the difference in quantitative variables distributions in independent samples. The Wilcoxon test for paired comparisons was used for dependent samples. Fisher’s exact test was used to test the difference in categorical variables. Null-hypothesis in statistical tests was rejected if p<0.05.

## Results

A complete standard neuropsychological examination and a battery of cognitive tests carried out along with the EAM technique at the first observation point (before radiation therapy) showed no evident impairments in patients’ cognitive sphere. Total results of tests assessing attention, memory, thinking processes, neurodynamic characteristics corresponded to standards. Slowly progression of a tumor compressing effect, which caused no destructions of brain macrostructures, was not accompanied by focal neuropsychological symptoms and deficit manifestations in the cognitive sphere. A total number of errors in free reproduction and recognition according to the EAM technique at the first observation point in the patients under study was no different from the number of errors recorded in the controls.

**Figure 2:**
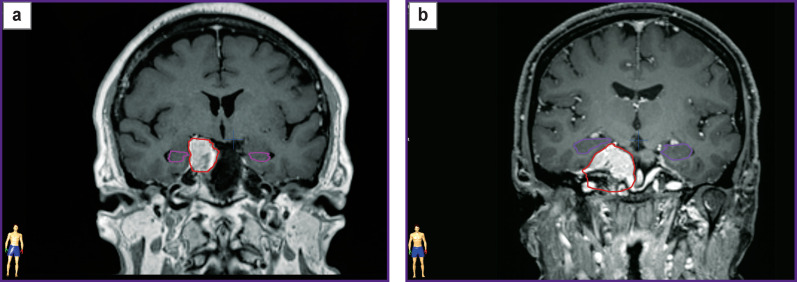
Topographometric MRI, coronal view, demonstrating the mechanical effect degree of the tumor on the hippocampus: (a) patient Z., the first subgroup, the hippocampuses are symmetric with respect to midline; (b) patient M., the second subgroup, the hippocampus on the tumor side is displaced upward and outside; *violet contours* — the hippocampus contouring; *red contours* — the tumor contouring

Based on MRI, the degree of a mechanical effect the tumor had on the temporal lobe and the hippocampus was determined in patients. Based on this characteristic, the sample was divided into two subgroups. The first subgroup included patients in whom the tumor compressed the temporal lobe, although the ipsilateral hippocampus was not displaced and was symmetrical to the contralateral hippocampus (Figure 2 (а)). The second subgroup consisted of the patients with compression of the hippocampus by the tumor, the latter being displaced and located asymmetrically relating to the contralateral one (Figure 2 (b)). In the end, a sampling of 28 patients with parasellar meningiomas was divided as follows: in 10 patients (5 with left-sided meningiomas and 5 with right-sided meningiomas), tumors were adjacent to the hippocampus but had no mechanical impact on the hippocampus (the first subgroup). In 18 patients (10 with left-sided meningiomas and 8 with right-sided meningiomas), the mass lesion compressed the hippocampus (the second subgroup). The subgroups appeared to be compatible in age, gender, education, and tumor location (p>0.05).

By a total number of free recall errors according to the EAM technique, the subgroups had no differences at the first point. After radiation therapy, which involuntarily affected a part of the hippocampus, some memory impairment was recorded in the subgroup with hippocampus compression: the median of the number of free recall errors increased from 4 [3; 6] at the first point to 6 [5; 9] at the second point (р=0.066). At the third point (6 months after therapy) positive changes were recorded in the subgroup without a mechanical effect on the hippocampus: the median of the number of recognition errors decreased from 8 [4; 12] at the first point to 6 [4; 8] at the third point (р=0.082).

**Figure 3:**
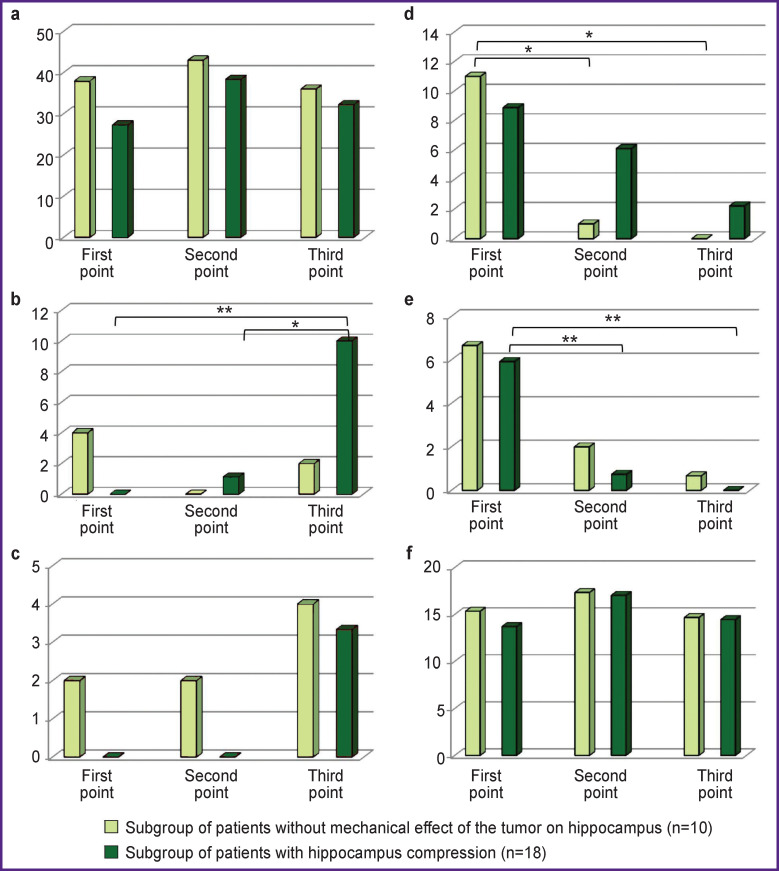
Dynamics of different errors in subgroups of patients with and without mechanic effect of the tumor on hippocampus: the errors neutralizing the novelty factor: (a) “similar/was”; (b) “was not/similar”; (c) “was not/was”; the errors enhancing a novelty factor: (d) “similar/was not”; (e) “was/was not”; (f) “was/similar”. Y-direction — average percentage of errors made in the subgroup. Significant differences: * p<0.05; ** p<0.01

However, the errors of recognition modeling the hippocampus functional activity were of greater interest for analysis. All possible errors according to the EAM technique are represented in the Table. Bar charts show the dynamics of the errors at observation points (Figure 3).

**Table:** Six possible errors during the recognition procedure (examples for one of the stimuli are represented)

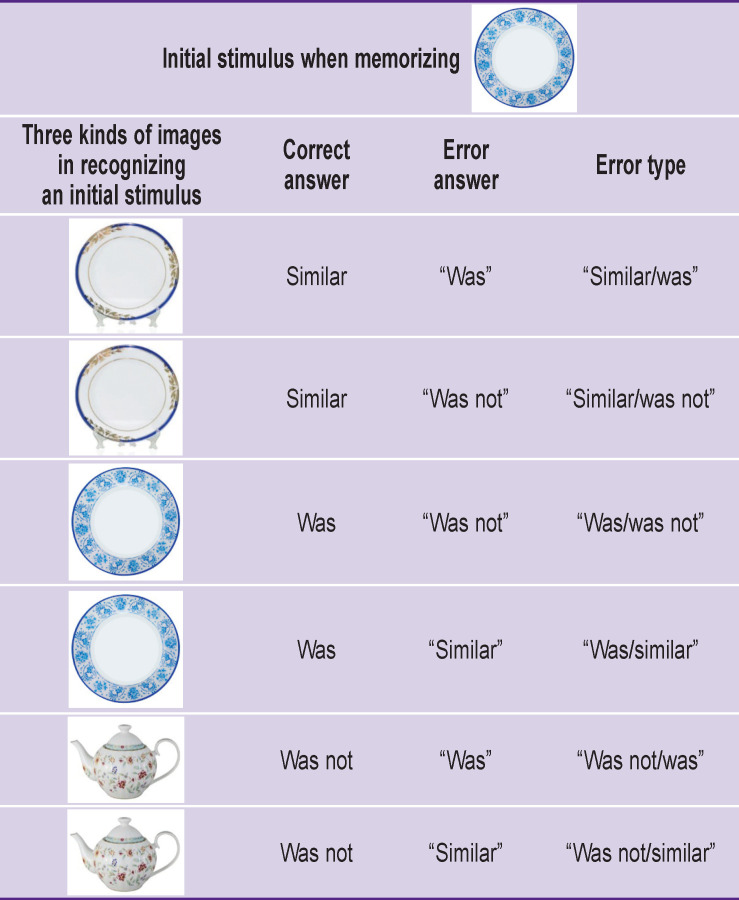

An error “similar/was” was the most common. Only this error type significantly prevailed in patients compared to healthy subjects (p=0.0007 — at the second point, and р=0.038 — at the third observation point).

Figure 3 (а) shows the dynamics of this error type in a longitudinal study. A significant increase in “similar/was” errors was recorded between the first and the second observation points (p=0.047), being more evident in the subgroup with a mechanical effect of the tumor on the hippocampus (р=0.055).

“Similar/was not” errors showed different dynamics. This error type regressed, to a larger degree, in the subgroup without the mechanical impact of the tumor on the hippocampus (the first subgroup), decreasing significantly at the second point (р=0.034). In contrast, no such errors were recorded at the third point (p=0.034).

There were no “was not/similar” errors in the second subgroup of patients at the first point. At the second point, there were few errors of this type, while at the third point, they differed significantly from those at the first point (р=0.008) and at the second point (р=0.025).

“Was/was not” errors in the subgroup with a mechanical effect on the hippocampus regressed significantly from point to point (p<0.01).

“Was not/was” errors somewhat grew in both subgroups by the third point, but due to the fact they were few, the tendency was insignificant.

Both groups had “was/similar” errors, the number of errors being the same compared to healthy subjects. They were not different in two subgroups and showed no dynamics from point to point.

## Discussion

The findings of the neuropsychological examination and the testing modeled to solve the assigned tasks confirmed our clinical observations that benign parasellar meningiomas are unlikely to be accompanied by marked cognitive deficiency and evident neuropsychological presentation due to their location and slow growth. A longitudinal study of the patients demonstrated that generally their cognitive condition was not deteriorating, and in a number of characteristics, it showed positive changes, probably due to the decrease in some tumor volume and the degree of its compression effect on adjacent structures after the therapy provided. The findings, on the one hand, confirm the relative safety of the applied radiotherapy protocol for the cognitive sphere, but on the other hand, they enable to study fine rearrangements in mental processes when the hippocampus is affected by two factors: soft compression by extracerebral lesion and the radiation factor, which can result in a complex of negative alterations in the brain (chronic inflammation, dystrophic and apoptotic changes in glial, endothelial and nervous cells) and have an inhibitory effect on neurogenesis in hippocampus [12, 13]. These two factors had their effect in different patients and at different time points. In addition, the first factor appeared to be significant only in the second subgroup of patients. The second factor was present only at the second and the third points.

Numerous medical reports [3, 4, 14, 15–17] represent indirect data on the hippocampus functional activity and its participation in information processing in humans. In general, its participation could be described as follows: a continuous flow of impressions occurring in an individual at any time of his active waking should be marked in a certain way. The resource-saving principle suggests that some part of impressions should be immediately forgotten (eliminated) as that already existing in the experience. A little part of the current experience should be taken as similar to that an individual has had before, although different by some important characteristics. And a minor part of impressions can be marked as principally new information of the current functioning. Impressions being marked at the hippocampus level cause different changes in the neuronal network. Further processing of continuous information streams depends on the value (old/similar/ new) assigned by the hippocampus. An experimental model of the EAM technique, to some extent, reproduces these complex aspects of hippocampus activity. Let us consider the study finding theoretically.

“Similar/was” error is an error a test subject makes when perceiving a similar image he cannot collate from memory, the features of the stimulus he had seen before, with the current one. The stimulus is marked as old, previously seen. This is the so-called pattern separation error. Differentiation of information patterns is rather rough. Precisely these errors are related to the deterioration of the hippocampus functional activity [14, 18, 19]. In the present study, at the first point, only these errors significantly prevailed in a clinical group compared to the healthy subjects. The fact can be considered as one more characteristic indicating the relation between such errors and the hippocampus. In dynamics, immediately after radiation therapy, the growth of “similar/was” errors was more significant in the second subgroup. It can be assumed that just a synchronizing action of two pathological factors — hippocampus compression and exposure to radiation — could result in such consequences.

Two relatively rare errors “was not/was” and “was not/similar” are incorrect marking of new stimuli as previously seen, old, or similar. Probably, these errors are of the same kind. However, in literature, they are not termed as pattern separation. The number of errors of both types also increased at the third point, being more evident in the patients with a mechanical impact of the tumor on the hippocampus.

The errors “was/was not” and “similar/was not” have another mechanism: here, on the contrary, an old stimulus marked as “old, has already been” is perceived as a new one, not seen before. Marking towards novelty is increasing. Both error types were regressing from point to point in both subgroups: the frequency of errors “was/was not” significantly decreased in the subgroup with a mechanical effect, the errors “similar/was not” — in the subgroup without a mechanical effect.

And, finally, the last error type — “was/similar”. This marking also increases the stimulus novelty: it is perceived as not an old one, already seen, but as a somewhat modified and having some distinguishing features. Both healthy subjects and the patients of both subgroups had this type of error, the frequency being equal. In addition, quantitatively, it did not change from point to point.

Thus, the patients with parasellar meningiomas during radiation therapy demonstrated the increase in the errors neutralizing the novelty factor in the stimuli presented, and on the contrary — there was a regress of the errors emphasizing the novelty factor. Such tendencies when a greater degree of differences between the stimuli was needed to assess two stimuli as similar rather than identical were recorded in decreasing neurogenesis in the hippocampus [6].

In general, the study showed that the errors in recognizing the stimuli related to the hippocampus functional activity can sensitively respond to different factors the hippocampus is exposed to; and the combination of two factors is accompanied by the more evident manifestation of these regularities. In addition, no decrease in the total efficiency of the cognitive and neuropsychological testing characteristics, safe social adaptation of the patients after radiotherapy suggests that benign extracerebral parasellar tumors exposed to 50–54 Gy in the mode of standard functioning do not cause significant cognitive changes deteriorating patients’ life quality.

## Conclusion

The factors of the hippocampus mechanical compression and its therapeutical radiation result in the growth of errors neutralizing the novelty of an information stream. And there is a reversed tendency for markings, which wrongly enhance stimuli novelty. The memory modulation factors reveal the fundamental role of the hippocampus in differentiating impressions of the current experience and can serve as the theoretical basis for subsequent effects on mnestic function.
